# Control of Glyphosate-Resistant Common Ragweed (*Ambrosia artemisiifolia* L.) in Glufosinate-Resistant Soybean [*Glycine max* (L.) Merr]

**DOI:** 10.3389/fpls.2017.01455

**Published:** 2017-08-18

**Authors:** Ethann R. Barnes, Stevan Z. Knezevic, Peter H. Sikkema, John L. Lindquist, Amit J. Jhala

**Affiliations:** ^1^Department of Agronomy and Horticulture, University of Nebraska-Lincoln Lincoln, NE, United States; ^2^Haskell Agricultural Laboratory, Northeast Research and Extension Center, University of Nebraska-Lincoln Concord, NE, United States; ^3^Department of Plant Agriculture, University of Guelph Ridgetown Ridgetown, ON, Canada

**Keywords:** gross profit margin, herbicide efficacy, residual herbicides, tank-mixture, weed resistance

## Abstract

Common ragweed emerges early in the season in Nebraska, USA and is competitive with soybean; therefore, preplant herbicides are important for effective control. Glyphosate has been used as a preplant control option; however, confirmation of glyphosate-resistant (GR) common ragweed in Nebraska necessitates evaluating other herbicide options. The objectives of this study were to (1) evaluate the efficacy of preplant (PP) herbicides followed by (fb) glufosinate alone or in tank-mixture with imazethapyr, acetochlor, or *S*-metolachlor applied post-emergence (POST) for control of GR common ragweed in glufosinate-resistant soybean; (2) their effect on common ragweed density, biomass, and soybean yield; and (3) the partial economics of herbicide programs. A field experiment was conducted in a grower's field infested with GR common ragweed in Gage County, Nebraska, USA in 2015 and 2016. Preplant herbicide programs containing glufosinate, paraquat, 2,4-D, dimethenamid-P, cloransulam-methyl, or high rates of flumioxazin plus chlorimuron-ethyl provided 90–99% control of common ragweed at 21 d after treatment (DAT). The aforementioned PP herbicides fb a POST application of glufosinate alone or in tank-mixture with imazethapyr, acetochlor, or *S*-metolachlor controlled GR common ragweed 84–98% at soybean harvest, reduced common ragweed density (≤20 plants m^−2^) and biomass by ≥93%, and secured soybean yield 1,819–2,158 kg ha^−1^. The PP fb POST herbicide programs resulted in the highest gross profit margins (US$373–US$506) compared to PP alone (US$91) or PRE fb POST programs (US$158). The results of this study conclude that effective and economical control of GR common ragweed in glufosinate-resistant soybean is achievable with PP fb POST herbicide programs.

## Introduction

Common ragweed (*Ambrosia artemisiifolia* L.) is a native, herbaceous, annual weed that belongs to the Asteracea family and is commonly found throughout temperate North America (Dickerson and Sweet, 1971; Coble et al., 1981). Common ragweed typically emerges early in the season in Nebraska, USA (Werle et al., 2014; Barnes et al., in press) and is a competitive weed in several agronomic crops, including soybean. Coble et al. (1981) reported that four common ragweed plants 10 m^−1^ row reduced 8% soybean yield. Similarly, Shurtleff and Coble (1985) and Weaver (2001) reported that 1.6 common ragweed plants m^−1^ row reduced soybean yield by 12 and 11%, respectively. Common ragweed is a monoecious species that has the potential to produce several thousand seeds per plant. A large (2.4 kg fresh weight) common ragweed plant can produce up to 62,000 seeds (Dickerson and Sweet, 1971) and can grow up to 2 m in height (Bassett and Crompton, 1975; Clewis et al., 2001). Allowing common ragweed seeds to enter the seed bank can lead to long term concern as seeds can remain viable in the soil for 39 years (Bassett and Crompton, 1975).

Glyphosate is a broad-spectrum, systemic, post-emergence (POST) herbicide (Duke and Powles, 2008) first marketed in 1974 (Franz et al., 1997). In 1996, GR soybean was first commercialized in the United States (Wiesbrook et al., 2001), and as of 2016, commercially grown GR crops include alfalfa (*Medicago sativa* L.), canola (*Brassica napus* L.), corn (*Zea mays* L.), cotton (*Gossypium hirsutum* L.), soybean (*Glycine max* L.), and sugarbeet (*Beta vulgaris* L.) (Duke and Powles, 2009). With the commercialization of GR crops, POST application of glyphosate increased dramatically (Dill, 2005), resulting in the evolution of GR weeds. As of 2016, glyphosate resistance has been reported in 37 weed species globally, including 16 species in the United States (Heap, 2017). Missouri was the first state to confirm GR common ragweed in 2004 (Pollard, 2007; Heap, 2017), and since then, GR common ragweed has been confirmed in 15 states in the United States and in Ontario, Canada (Heap, 2017). GR common ragweed has been recently confirmed as the sixth GR weed in Nebraska, USA (Ganie and Jhala, 2017). In response to widespread adoption of GR corn and soybean, and the effective, broad-spectrum, and affordable weed control with glyphosate, no-tillage, and reduced tillage production systems increased as the use of glyphosate replaced pre-plant tillage (Givens et al., 2009). No-till soybean production reduces soil erosion and operating cost while providing comparable yields to conventional tillage systems (Stougaard et al., 1984).

Glufosinate blocks the glutamine synthetase enzyme, which leads to buildup of ammonium in plant tissue (Logusch et al., 1991). Glufosinate is a broad-spectrum, contact herbicide (Haas and Muller, 1987). Glufosinate-resistant soybean was first commercialized in 1999 (Wiesbrook et al., 2001). It can be applied up to 1,329 g ai ha^−1^ per growing season in glufosinate-resistant soybean in either single or sequential (>5 d apart) application up to but not including the bloom soybean growth stage (Anonymous, 2016). Glufosinate has no plant-back interval for corn or soybean and can be applied in a range of 593–736 g ai ha^−1^ in a single application depending on weed pressure (Anonymous, 2016). Glufosinate is an alternative herbicide option for control of GR weeds in glufosinate-resistant soybean if applied as per label direction (Jhala et al., 2014; Kaur et al., 2014).

Management of GR weeds is a challenge for soybean producers in Nebraska. Widespread occurrence of GR weeds in several states in the Midwestern United States, including Nebraska, requires alternate weed management programs. Planting of glufosinate-resistant soybean is increasing in several states, specifically for control of GR weeds. A survey conducted in 2011 in Arkansas reported that 12% of the soybean acreage was seeded to glufosinate-resistant cultivars (Riar et al., 2013), a number that had increased to 35% by 2016 (JK Norsworthy, personal communication). Similarly, the use of glufosinate-resistant soybean cultivars has increased in recent years in the Midwest (Jhala et al., 2017).

Preplant application of 2,4-D, flumioxazin, glufosinate, paraquat, saflufenacil, or sulfentrazone followed by (fb) a POST application of glufosinate alone or in tank-mixtures effectively controlled GR giant ragweed (*Ambrosia trifida* L.), a closely related species of common ragweed, in Nebraska (Kaur et al., 2014). Aulakh and Jhala (2015) reported that sulfentrazone plus metribuzin applied PRE fb a POST application of glufosinate tank-mixed with acetochlor, pyroxasulfone, or *S*-metolachlor controlled common lambsquarters (*Chenopodium album* L.), common waterhemp (*Amaranthus rudis* Sauer), eastern black nightshade (*Solanum ptychanthum* Dunal), velvetleaf (*Abutilon theophrasti* Medik.), large crabgrass (*Digitaria sanguinalis* L.), and green foxtail (*Setaria viridis* L.) ≥90% in glufosinate-resistant soybean. Van Wely et al. (2014, 2015) concluded that neither a single PP nor a single POST herbicide application provided full season control of GR common ragweed in GR soybean in Ontario and that PP fb POST programs would need to be considered. Common ragweed's early emergence reduces the likelihood of controlling it with a PRE application as most common ragweed have already emerged. The control of summer emerging weeds such as common waterhemp or Palmer amaranth (*Amaranthus palmeri S. Wats*.) requires the use of a residual PRE herbicide for control (Oliveira et al., 2017; Sarangi et al., 2017).

There has been no study published in the scientific literature about control of GR common ragweed in glufosinate-resistant soybean. The objectives of this study were to evaluate the efficacy of PP herbicides fb glufosinate alone or in tank-mixture with acetochlor, imazethapyr, or *S*-metolachlor for control of GR common ragweed in glufosinate-resistant soybean, their effect on soybean injury and yield, and the economics of herbicide programs. The hypothesis for this study was that a PP application of an effective herbicide fb glufosinate will provide effective control of GR common ragweed in glufosinate-resistant soybean.

## Materials and methods

Field experiments were conducted in Gage County, Nebraska, USA in 2015 and 2016 in a field with confirmed GR common ragweed infestation (Ganie and Jhala, 2017). The field was non-irrigated and in a corn-soybean rotation which was planted to corn in 2014 and soybean in 2015. The research site consisted of a Wymore silty clay loam (37.6% silt, 37.6% clay, and 24.8% sand) with 2.5% organic matter and a pH of 6.0. The experimental design was a randomized complete block with 14 treatments (Table 1) and four replications. The plot size was 3 m wide (4 soybean rows spaced 0.75 m apart) by 9 m in length. Glufosinate-resistant soybean (5290LL, NuPride Genetics Network P.O. Box 830911 Lincoln, NE 68583) was planted under no-tillage conditions on May 19, 2015 and May 26, 2016 at a population of 300,000 seeds ha^−1^ to a depth of 3 cm. The experiments included 13 herbicide programs comprised of four application timings: preplant (PP), pre-emergence (PRE), early POST (EPOST), and late POST (LPOST) (Table 1). The field experiments were conducted at grower's field infested with glyphosate-resistant common ragweed. The grower's field had limited space available to conduct research projects. Therefore, the treatment list was restricted and a weed-free control was not included. POST applications were scheduled based on soybean growth stage with EPOST applied around the third soybean trifoliate and LPOST applied before soybean began flowering. For comparison, a non-treated control was included. The labeled rate of each herbicide was used for all treatments.

**Table 1:** Herbicide treatments, application timing and rate, and products used in a field experiment conducted in Gage County, NE in 2015 and 2016.

**Herbicide program**	**Timing^a^**	**Rate**	**Trade name**	**Manufacturer^b^**	**Adjuvant^c^**
		g ai ha^−1^			
Glufosinate	PP	594	Liberty 280	Bayer	AMS
Saflufenacil + Imazethapyr +Dimethenamid-P fb Glufosinate	PP EPOST	95 + 1,100 740	Optill + Outlook fb Liberty 280	BASF + BASF fb Bayer	MSO + AMS AMS
Sulfentrazone + Cloransulam-methyl fb Glufosinate	PP EPOST	314 740	Authority First fb Liberty 280	FMC fb Bayer	COC + AMS AMS
Flumioxazin + Chlorimuron-ethyl fb Glufosinate	PP EPOST	140 740	Valor XLT fb Liberty 280	Valent fb Bayer	COC + AMS AMS
S-metolachlor + Metribuzin fb Glufosinate	PP EPOST	2,050 740	Boundary fb Liberty 280	Syngenta fb Bayer	COC + AMS AMS
Chlorimuron-ethyl + Flumioxazin +Thifensulfuron-methyl fb Glufosinate	PP EPOST	94 740	Envive fb Liberty 280	Dupont fb Bayer	COC + AMS AMS
2,4-D fb Glufosinate + Imazethapyr	PP EPOST	1,180 740 + 70	2,4-D Amine fb Liberty 280 + Pursuit	Winfield fb Bayer + BASF	NIS + AMS NIS + AMS
Paraquat fb Glufosinate + Chlorimuron-ethyl + Acetochlor	PP EPOST	1,120 740 + 13.1 + 1,680	Gramoxone Inteon fb Liberty 280 + Classic + Warrant	Syngenta fb Bayer + Dupont + Monsanto	COC NIS + AMS
Saflufenacil fb Glufosinate + Acetochlor	PP EPOST	150 740 + 1,680	Sharpen fb Liberty 280 + Warrant	BASF fb Bayer + Monsanto	MSO + AMS AMS
Saflufenacil + 2,4-D fb Glufosinate + Acetochlor	PP EPOST	150 + 1,180 740 + 1,680	Sharpen + 2,4-D Amine fb Liberty 280 + Warrant	BASF + Winfield fb Bayer + Monsanto	MSO + AMS AMS
Flumioxazin + Chlorimuron-ethyl fb Glufosinate + S-metolachlor fb Glufosinate + Acetochlor	PP EPOST LPOST	112 594 + 1,480 594 + 1,260	Valor XLT fb Liberty 280 fb Warrant	Valent fb Bayer fb Monsanto	COC + AMS NIS + AMS AMS
2,4-D fb Sulfentrazone + Metribuzin fb Glufosinate	PP PRE LPOST	1,180 5.7 740	2,4-D Amine fb Authority MTZ fb Liberty 280	Winfield fb FMC fb Bayer	NIS + AMS COC AMS
Sulfentrazone + Metribuzin fb Glufosinate	PRE LPOST	6.3 740	Authority MTZ fb Liberty 280	FMC fb Bayer	COC AMS

a *AMS, ammonium sulfate (DSM Chemicals orth America Inc., Augusta, GA); COC, crop oil concentrate (Agridex, Helena Chemical Co., Collierville, TN); PP, Preplant; EPOST, early POST; LPOST, late POST; fb, followed by; MSO, methylated seed oil (Southern Ag Inc., Suwanee, GA); NIS, nonionic surfactant (Induce, Helena Chemical Co., Collierville, TN)*.

b *Bayer CropScience, Research Triangle Park, NC 27709; Valent U.S.A. Corporation, Walnut Creek, CA 94596; FMC Corporation, Philadelphia, PA 19103; BASF Corporation, Research Triangle Park, NC 27709; Syngenta Crop Protection, Greensboro, NC 27419; Monsanto Company, St. Louis, MO 63167; DuPont Crop Protection, P.O. Box 80705, Wilmington, DE 19880; Winfield Solutions, LLC, P.O. Box 64589, St. Paul, MN 55164-0589*.

c *AMS at 2% (wt/v), COC or MSO at 1% (v/v), and NIS at 0.25% (v/v) were mixed with herbicides*.

Herbicides were applied with a CO_2_ pressurized backpack sprayer and a boom equipped with four TT 110015 flat-fan nozzles (TeeJet, Spraying Systems Co., P.O. Box 7900, Wheaton, IL 60189) spaced 60 cm apart. Treatments were applied as PP (May 1, 2015 and May 5, 2016), PRE (May 21, 2015 and May 26, 2016), EPOST (June 16, 2015 and June 16, 2016), and LPOST (July 17, 2015 and June 30, 2016). Common ragweed ranged from 1–8 cm tall at the time of PP, 4–16 cm at PRE, 16–45 cm at EPOST, and 36–60 cm at the time of LPOST. Common ragweed control was assessed visually on a scale of 0–100%, with 0% representing no control and 100% representing complete control, at 21 d after PP and PRE, 14 DAEPOST and LPOST, and at soybean harvest. Soybean injury was assessed on a scale of 0–100%, with 0% representing no injury and 100% representing plant death, at 21 DAPRE, and 14 DAEPOST and LPOST. Common ragweed densities were assessed from two randomly placed 0.25 m^2^ quadrats in each plot at 7 DAPRE, 14 DAEPOST, and 14 DALPOST. Common ragweed aboveground biomass was assessed from two randomly placed 0.25 m^2^ quadrat in each plot at 70 DALPOST. Surviving common ragweed plants were cut near the soil surface, dried in paper bags at 50 C for 10 d, and their biomass was recorded. Percent biomass reduction compared with the non-treated control was calculated using the equation (Wortman, 2014):

(1) % Biomass reduction=[(C−B)/C]×100

where *C* represents the common ragweed biomass from the non-treated control plot in the corresponding replication block and *B* represents the biomass of the treatment plots. Soybean was harvested with a plot combine and the yields were adjusted to 13% moisture content. Gross profit margin was calculated as gross revenue minus herbicide and application costs (Norsworthy and Oliver, 2001). Average herbicide prices from three independent commercial sources (Cargill, Country Partners Cooperative, Crop Production Services) in Nebraska were used to calculate herbicide cost ha^−1^. Herbicide program cost was calculated by summing the herbicide cost ha^−1^ for each treatment and adding a custom application cost of US$18.11 ha^−1^ application^−1^, the average of the three aforementioned independent sources in Nebraska. Gross revenue was calculated from the average yield for each treatment based on the average price received in Nebraska during harvest time in 2015 and 2016 (US$0.33 kg^−1^; USDA, 2016).

### Statistical analysis

Data were subjected to ANOVA using PROC GLIMMIX procedure in SAS version 9.3 (SAS Institute Inc., Cary, NC). Years and treatments were considered fixed effects and replications nested within year were considered random effects in the model. Data were tested for normality using PROC UNIVARIATE before analysis. An arcsine square-root transformation was performed on common ragweed control estimates and biomass reduction data before analysis; however, data were back-transformed for presentation of results. Treatment means were separated at *P* ≤ 0.05 using Fisher's protected least significant difference test. Orthogonal contrasts were conducted to compare PP fb POST treatments vs. PP alone, PRE fb LPOST, or PP fb PRE fb LPOST treatments.

## Results

Year-by-treatment interactions for GR common ragweed control, density, biomass, and soybean yield were not significant; therefore, data were combined. Average daily temperatures during the study were similar to the 30-year average (Table 2). May and June of 2015 received higher precipitation (36.2 cm) than the 30-year average (18.6 cm); however, the 2016 growing season received similar precipitation to the 30-year average (16.3 cm in May and June; Table 2).

**Table 2:** Average monthly temperature and precipitation in a field experiment conducted in Gage County, NE in 2015 and 2016^a^^,^ ^b^.

	**Temperature (C)**			**Precipitation (cm)**		
	**2015**	**2016**	**30 yr avg**	**2015**	**2016**	**30 yr avg**
May	15.5	15.6	16.1	11.7	7.0	6.3
June	21.6	23.4	21.7	24.5	9.3	12.3
July	23.6	22.4	24.3	13.2	6.4	11.0
August	21.5	23.0	23.6	9.2	9.1	10.2
September	21.6	20.3	18.8	6.0	12.2	9.0

a *30 yr avg, 30 year average (1981–2010)*.

b *Monthly weather data acquired from the nearest High Plains Regional Climate Center station near Virginia, NE*.

### Common ragweed control

Most of the PP herbicides controlled GR common ragweed ≥90% at 21 DAPP (Table 3). For example, herbicide programs containing glufosinate, paraquat, 2,4-D, imazethapyr, cloransulam-methyl, and flumioxazin provided 90–99% control of common ragweed at 21 DAPP (Table 3). A premix of flumioxazin and chlorimuron-ethyl provided 93–96% control at 14 DAPP in this study. Saflufenacil controlled common ragweed 75% at 21 DAPP; however, tank-mixing with imazethapyr plus dimethenamid-P as well as with 2,4-D provided 97 and 99% control, respectively (Table 3). Among PP herbicide programs, chlorimuron-ethyl plus flumioxazin plus thifensulfuron-methyl resulted in the lowest (52%) common ragweed control at 21 DAPP.

**Table 3:** Orthogonal contrasts for comparison of herbicide programs and control of glyphosate-resistant common ragweed in glufosinate-resistant soybean at 21 DAPP, 21 DAPRE, 14 DAEPOST, 14 DALPOST, and at harvest in a field experiment conducted in Gage County, NE in 2015 and 2016^a^.

**Herbicide program**	**Timing**	**Rate**	**Common ragweed control^b^^,^^c^**				
			**21 DA PP(%)**	**21 DA PRE(%)**	**14 DA EPOST(%)**	**14 DA LPOST(%)**	**At Harvest(%)**
		g ai ha^−1^					
Glufosinate	PP	594	92 ab	81 bc	40 d	0 f	0 e
Saflufenacil + Imazethapyr +Dimethenamid-P fb Glufosinate	PP EPOST	95 + 1,100 740	97 a	94 ab	97 abc	97 ab	97 abc
Sulfentrazone + Cloransulam-methyl fb Glufosinate	PP EPOST	314 740	96 a	91 ab	97 abc	97 ab	96 abc
Flumioxazin + Chlorimuron-ethyl fb Glufosinate	PP EPOST	140 740	93 a	84 abc	96 abc	96 abc	84 bc
S-metolachlor + Metribuzin fb Glufosinate	PP EPOST	2,050 740	76 b	70 cd	92 bc	91 de	87 abc
Chlorimuron-ethyl + Flumioxazin + Thifensulfuron-methyl fb Glufosinate	PP EPOST	94 740	52 c	45 e	91 c	88 e	62 d
2,4-D fb Glufosinate + Imazethapyr	PP EPOST	1,180 740 + 70	90 ab	88 abc	98 ab	97 ab	96 abc
Paraquat fb Glufosinate + Chlorimuron-ethyl + Acetochlor	PP EPOST	1,120 740 + 13.1 + 1,680	96 a	80 bc	96 abc	95 bcd	94 abc
Saflufenacil fb Glufosinate + Acetochlor	PP EPOST	150 740 + 1,680	75 b	55 de	93 bc	91 cde	64 d
Saflufenacil + 2,4-D fb Glufosinate + Acetochlor	PP EPOST	150 + 1,180 740 + 1,680	99 a	98 a	99 a	99 a	98 ab
Flumioxazin + Chlorimuron-ethyl fb Glufosinate + S-metolachlor fb Glufosinate + Acetochlor	PP EPOST LPOST	112 594 + 1,480 594 + 1,260	96 a	92 ab	99 a	99 a	99 a
2,4-D fb Sulfentrazone + Metribuzin fb Glufosinate	PP PRE LPOST	1,180 5.7 740	95 a	97 a	93 bc	99 a	99 ab
Sulfentrazone + Metribuzin fb Glufosinate	PRE LPOST	6.3 740	0 c	18 f	9 e	92 e	88 c
*P*-value			<0.0001	<0.0001	<0.0001	<0.0001	<0.0001
**ORTHOGONAL CONTRASTS^d^**							
PP vs. PRE			–	80 vs. 18^****^	–	–	–
PP fb EPOST vs. PP only			–	–	95 vs. 40^****^	95 vs. 0^****^	86 vs. 0^****^
PP fb EPOST vs. PRE fb LPOST			–	–	95 vs. 9^****^	95 vs. 92 ns	86 vs. 88 ns
PP fb EPOST vs. PP fb PRE fb LPOST			–	–	95 vs. 93 ns	95 vs. 99^**^	86 vs. 99 ^*^
PP fb EPOST vs. PP fb EPOST fb LPOST			–	–	–	95 vs. 99^**^	86 vs. 99^*^

a *DA, days after; EPOST, early POST; fb, followed by; LPOST, late POST; PP, Preplant*.

b *Year by treatment interaction was not significant; therefore, data from both years were combined. Data were arc-sine square-root transformed before analysis; however, back transformed values are presented based on the interpretation from the transformed data*.

c *Means presented within the same column with no common letter(s) are significantly different according to Fisher's Protected LSD where α = 0.05*.

d Significance levels: ns, non-significant;

* P < 0.05;

** P < 0.01;

^***^ P < 0.001;

**** *P < 0.0001*.

A PRE application of sulfentrazone plus metribuzin following a PP application of 2,4-D controlled GR common ragweed 97% at 21 DAPRE, comparable with several other treatments with only PP application; however, when sulfentrazone plus metribuzin was applied PRE even at a higher rate (6.3 g ai ha^−1^) without a PP herbicide application, it resulted in 18% control of common ragweed at 21 DAPRE (Table 3). Moreover, the contrast statement confirmed that PP applications controlled 80% of common ragweed compared to a PRE application that resulted in only 18% control at 21 DAPRE (Table 3).

The PP herbicides fb glufosinate EPOST, alone or in tank-mixtures, controlled common ragweed 91–99% at 21 DAEPOST (Table 3). A LPOST application of glufosinate following a PP application of 2,4-D and a PRE application of sulfentrazone plus metribuzin controlled GR common ragweed 99% at 14 DALPOST. Glufosinate LPOST following sulfentrazone plus metribuzin PRE controlled GR common ragweed 92%. Glufosinate plus acetochlor applied LPOST following an EPOST application of glufosinate plus *S*-metolachlor and a PP application of flumioxazin plus chlorimuron-ethyl controlled GR common ragweed 99% at 14 DALPOST, comparable with several PP fb EPOST programs.

Most of herbicide programs that included both a PP and POST herbicide application provided season-long control (≥87%) of common ragweed at harvest (Table 3). Herbicide programs including chlorimuron-ethyl plus flumioxazin plus thifensulfuron-methyl or saflufenacil applied PP fb glufosinate EPOST, alone or tank-mixed with acetochlor, controlled GR common ragweed 62–64% at harvest. A single PP application of glufosinate controlled GR common ragweed 0% at harvest, suggesting that an in-crop application is needed for season-long common ragweed control. Sulfentrazone plus metribuzin applied PRE fb glufosinate applied LPOST controlled GR common ragweed 88% at harvest; however, when a PP application of 2,4-D was added to the program, the control increased to 99%. Orthogonal contrasts conclude that PP and PP fb EPOST herbicide programs controlled GR common ragweed 0 and 86% at harvest, respectively, and PP fb PRE fb LPOST program controlled GR common ragweed 99% at harvest (Table 3).

### Common ragweed density and biomass

Common ragweed density for the non-treated control was 1,337 and 1,159 plants m^−2^ at 7 DAPRE and 14 DAEPOST, respectively, compared with the average of herbicide treatments (305 and 177 plants m^−2^, respectively; Table 4). Preplant herbicides resulted in common ragweed densities of 0 to 366 plants m^−2^, except saflufenacil (844 plants m^−2^), and chlorimuron-ethyl plus flumioxazin plus thifensulfuron-methyl (1,180 plants m^−2^; Table 4). The PP application of 2,4-D fb sulfentrazone plus metribuzin applied PRE reduced common ragweed density to eight plants m^−2^ at 7 DAPRE compared with sulfentrazone plus metribuzin applied PRE without PP herbicide application (908 plants m^−2^; Table 4).

**Table 4:** Orthogonal contrasts for comparison of herbicide programs and effect of herbicide programs on glyphosate-resistant common ragweed density at 7 DAPRE, 14 DAEPOST, and 14 DALPOST, common ragweed biomass reduction, and soybean yield in a field experiment conducted in Gage County, NE in 2015 and 2016^a^.

**Herbicide**	**Timing**	**Rate**	**Common ragweed density^b^**			**Biomass reduction^b^^,^^c^**	**Soybean yield^b^**
			**7 DA PRE**	**14 DA EPOST**	**14 DA LPOST**		
		g ai ha^−1^		Plants m^−2^		%	kg ha^−1^
Nontreated control			1,337 a	1,159 a	1,145^d^	0 e	32 c
Glufosinate	PP	594	159 c	120 c	101 a	14 e	474 c
Saflufenacil + Imazethapyr +Dimethenamid-P fb Glufosinate	PP EPOST	95 + 1,100 740	11 c	0 c	0 d	98 abcd	2,158 a
Sulfentrazone + Cloransulam-methyl fb Glufosinate	PP EPOST	314 740	67 c	17 c	15 cd	100 ab	1,922 a
Flumioxazin + Chlorimuron-ethyl fb Glufosinate	PP EPOST	140 740	221 c	18 c	20 bcd	93 abcd	1,897 a
S-metolachlor + Metribuzin fb Glufosinate	PP EPOST	2,050 740	366 c	57 c	56 abc	86 abcd	1,819 a
Chlorimuron-ethyl + Flumioxazin + Thifensulfuron-methyl fb Glufosinate	PP EPOST	94 740	1,180 ab	108 c	102 a	82 d	1,860 a
2,4-D fb Glufosinate + Imazethapyr	PP EPOST	1,180 740 + 70	88 c	6 c	8 cd	99 abc	1,899 a
Paraquat fb Glufosinate + Chlorimuron-ethyl + Acetochlor	PP EPOST	1,120 740 + 13.1 + 1,680	59 c	3 c	0 d	93 abcd	1,859 a
Saflufenacil fb Glufosinate + Acetochlor	PP EPOST	150 740 + 1,680	844 b	62 c	68 ab	81 cd	1,819 a
Saflufenacil + 2,4-D fb Glufosinate + Acetochlor	PP EPOST	150 + 1,180 740 + 1,680	0 c	0 c	0 d	100 a	2,115 a
Flumioxazin + Chlorimuron-ethyl fb Glufosinate + S-metolachlor fb Glufosinate + Acetochlor	PP EPOST LPOST	112 594 + 1,480 594 + 1,260	51 c	0 c	0 d	100 a	2,003 a
2,4-D fb Sulfentrazone + Metribuzin fb Glufosinate	PP PRE LPOST	1,180 5.7 740	8 c	7 c	0 d	100 a	2,060 a
Sulfentrazone + Metribuzin fb Glufosinate	PRE LPOST	6.3 740	908 ab	744 b	93 a	83 bcd	1,014 b
*P*-value			<0.0001	<0.0001	<0.0001	<0.0001	<0.0001
**ORTHOGONAL CONTRASTS^e^**							
PP vs. PRE			277 vs. 908^***^	–	–	–	–
PP fb EPOST vs. PP only			–	30 vs. 120ns	30 vs. 101^***^	92 vs. 14^****^	1,928 vs. 474^****^
PP fb EPOST vs. PRE fb LPOST			–	30 vs. 744^****^	30 vs. 93^**^	92 vs. 83 ns	1,928 vs. 1,014^****^
PP fb EPOST vs. PP fb PRE fb LPOST			–	30 vs. 7 ns	30 vs. 0 ns	92 vs. 100 ns	1,928 vs. 2,060 ns
PP fb EPOST vs. PP fb EPOST fb LPOST			–	–	30 vs. 0 ns	92 vs. 100 ns	1,928 vs. 2,003 ns

a *DA, days after; EPOST, early POST; fb, followed by; LPOST, late POST; PP, preplant; vs., versus*.

b *Means presented within the same column with no common letter(s) are significantly different according to Fisher's Protected LSD where α = 0.05*.

c *Year by treatment interaction was not significant; therefore, data from both years were combined. Data were arc-sine square-root transformed before analysis; however, back transformed values are presented based on the interpretation from the transformed data*.

d *Nontreated control was excluded from analysis as an outlier*.

e Significance levels: ns, non-significant;

^*^ P < 0.05;

** P < 0.01;

*** P < 0.001;

**** *P < 0.0001*.

Based on orthogonal contrasts, PP and PRE herbicide programs on average resulted in 277 and 908 plants m^−2^ at 7 DAPRE, respectively (Table 4). The PP fb PRE fb LPOST or PP fb EPOST fb LPOST did not reduce common ragweed densities compared to PP fb EPOST programs. Averaged across treatments, PP fb EPOST program had lower common ragweed density (30 plants m^−2^) compared with PP (101 plants m^−2^) or PRE fb LPOST (93 plants m^−2^) program at 14 DALPOST (Table 4). Most herbicide programs with PP application resulted in 81–100% common ragweed biomass reduction. Averaged across treatments, PP, PP fb EPOST, and PRE fb LPOST reduced GR common ragweed biomass 14, 92, and 83%, respectively.

### Soybean yield

The lowest soybean yield was obtained in the non-treated control (32 kg ha^−1^) and with glufosinate applied PP (474 kg ha^−1^). Herbicide programs that included an effective PP fb glufosinate applied POST, alone or in tank-mixture, resulted in soybean yields 1,819–2,158 kg ha^−1^ with no difference among them (Table 4). Averaged across treatments, PP fb EPOST programs resulted in higher yields (1,928 kg ha^−1^) compared with PP alone (474 kg ha^−1^) or PRE fb LPOST (1,014 kg ha^−1^) herbicide programs (Table 4). Averaged across treatments, PP fb EPOST programs resulted in similar yields (1,928 kg ha^−1^) compared with PP fb PRE fb EPOST (2,060 kg ha^−1^) or PP fb EPOST fb LPOST (2,003 kg ha^−1^); therefore, if common ragweed is the major weed in a soybean field, a PP fb EPOST program can provide full season control and PP fb PRE fb POST programs are not needed to achieve optimum soybean yield (Table 4).

### Economics

The cost of PP fb POST herbicide programs ranged from US$131.30 to $257.87 ha^−1^ and provided maximum gross profit margins (Table 5). The PP application of saflufenacil plus imazethapyr plus dimethenamid-P fb glufosinate EPOST cost $197.37 ha^−1^ and resulted in the highest gross profit margin of $505.96 ha^−1^ (Table 5). Glufosinate applied PP alone had the lowest cost ($63.25 ha^−1^); but resulted in a gross profit margin of only $91.23 ha^−1^ due to poor control of common ragweed that resulted in low soybean yield (Table 5). The PRE fb LPOST program resulted in a gross profit margin of $158.23 ha^−1^ (Table 5). Although the PP fb PRE fb POST program resulted in 99% common ragweed control and 2,060 kg ha^−1^ soybean yield, gross profit margin was $471.14 compared with $372.79 to $505.96 for PP fb POST programs due to additional cost of PRE herbicide and application.

**Table 5:** Cost of herbicide programs for controlling glyphosate-resistant common ragweed in glufosinate-resistant soybean, income from soybean yield, and gross profit margin in a field experiment conducted in Gage County, NE in 2015 and 2016.^a^

**Herbicide**	**Timing**	**Rate**	**Program cost^b^**	**Gross revenue from soybean yield^c^**	**Gross profit margin^d^**
		g ai ha^−1^		$ ha^−1^	
Nontreated control	–	–	0.00	10.43	10.43
Glufosinate	PP	594	63.25	154.48	91.23
Saflufenacil + Imazethapyr + Dimethenamid-P fb Glufosinate	PP EPOST	95 + 1,100 740	197.37	703.33	505.96
Sulfentrazone + Cloransulam-methyl fb Glufosinate	PP EPOST	314 740	180.21	626.41	446.20
Flumioxazin + Chlorimuron-ethyl fb Glufosinate	PP EPOST	140 740	168.33	618.26	449.94
S-metolachlor + Metribuzin fb Glufosinate	PP EPOST	2,050 740	155.21	592.84	437.63
Chlorimuron-ethyl + Flumioxazin + Thifensulfuron-methyl fb Glufosinate	PP EPOST	94 740	131.30	606.20	474.91
2,4-D fb Glufosinate + Imazethapyr	PP EPOST	1,180 740 + 70	146.20	618.92	472.71
Paraquat fb Glufosinate + Chlorimuron-ethyl + Acetochlor	PP EPOST	1,120 740 + 13.1 + 1,680	176.30	605.88	429.57
Saflufenacil fb Glufosinate + Acetochlor	PP EPOST	150 740 + 1,680	220.05	592.84	372.79
Saflufenacil + 2,4-D fb Glufosinate + Acetochlor	PP EPOST	150 + 1,180 740 + 1,680	230.01	689.31	459.30
Flumioxazin + Chlorimuron-ethyl fb Glufosinate + S-metolachlor fb Glufosinate + Acetochlor	PP EPOST LPOST	112 594 + 1,480 594 + 1,260	257.87	652.81	394.94
2,4-D fb Sulfentrazone + Metribuzin fb Glufosinate	PP PRE LPOST	1,180 5.7 740	200.25	671.39	471.14
Sulfentrazone + Metribuzin fb Glufosinate	PRE LPOST	6.3 740	172.25	330.48	158.23

a *Herbicide costs were averaged from three independent sources in Nebraska*.

b *Program cost includes an average cost of application ($18.11 ha^−1^ application^−1^) from three independent sources in Nebraska*.

c *Gross Revenue from soybean yield was based on an average price received in Nebraska on the harvest month*.

d *Gross profit margins were calculated as gross revenue from soybean yield minus program cost*.

## Discussion

Six GR weeds, including common ragweed, have been confirmed in Nebraska and their management is a challenge for crop producers. This is the first report describing control of GR common ragweed in glufosinate-resistant soybean. Common ragweed is an early emerging weed in Nebraska. It has been reported that common ragweed start emerging in March reaching 10% emergence around 259 growing degree day (GDD) and 90% of emergence is achieved by the first or second week of May or 757 GDD calculated with a base temperature of 3 C (Barnes et al., in press). Shrestha et al. (1999) reported the base temperature for common ragweed to be 3.6 C. Therefore, as observed in this study, preplant application of herbicide is critical for control of GR common ragweed. This agrees with the findings of Kaur et al. (2014) and Jhala et al. (2014) reporting that PP application of herbicide is important for control of GR giant ragweed in Nebraska. Similarly, Ganie et al. (2016) reported that GR giant ragweed control was reduced to <83% at 21 DAPRE and ≤78% at harvest when PP herbicides were not included in the program.

Results of this study reported that a number of herbicide options such as saflufenacil plus imazethapyr plus dimethenamid-P, suflentrazone plus cloransulam-methyl, paraquat, safluefenacil plus 2,4-D, 2,4-D, and flumioxazin plus chlorimuron-ethyl are available for common ragweed control. Control of 1–8 cm tall common ragweed with PP herbicides in this study was similar to that which was reported in the literature. For example, Corbett et al. (2004) reported ≥99% control of 2–10 cm tall common ragweed at 14 and 20 DAT with glufosinate. Additionally, 83–85% common ragweed control was reported at 14 DAT with cloransulam-methyl (Taylor et al., 2002). Wilson and Worsham (1988) reported 83 and 64% common ragweed control at 28 DAT from paraquat and 2,4-D, respectively, with half the rates used in this study. Niekamp et al. (1999) reported 98% common ragweed control at 7 DAT with flumioxazin (90 g ai ha^−1^) and chlorimuron-ethyl (70 g ai ha^−1^). Kaur et al. (2014) further reported 96% control of giant ragweed with saflufenacil applied alone and 99% control when tank-mixed with 2,4-D at 14 DAT.

Preplant herbicide was followed by PRE and/or POST herbicide application for season-long control of GR common ragweed. A follow up application after PP was needed to avoid poor control and potential yield reduction. Glufosinate applied POST alone or in a tank-mixture with imazethapyr, acetochlor, or *S*-metolachlor controlled GR common ragweed 84–98%. Tharp and Kells (2002) reported that PRE herbicide followed by glufosinate controlled common ragweed, redroot pigweed (*Ameranthus retroflexus* L.), and common lambsquarters ≥92% at 28 DAT. Tharp and Kells (2002) also reported that glufosinate tank-mixed with *S*-metolachlor or acetochlor controlled common ragweed ≥99% at 28 DAT. Although a PP application fb sequential POST applications provided 99% control at 14 DALPOST, statistically it was comparable to PP fb single POST programs indicating that an effective PP herbicide fb a single POST application of glufosinate controlled GR common ragweed >90% and that a second POST application is not needed.

Preplant fb POST herbicide programs on average resulted in less common ragweed density (30 plants m^−2^) and greater biomass reductions (92%) than single applications which is consistent with the literature. For instance, Aulakh and Jhala (2015) reported ≤4 plants m^−2^ for common lambsquarters, common waterhemp, eastern black nightshade, and velvetleaf; and ≤2 plants m^−2^ for green foxtail and large crabgrass at harvest with the use of PRE fb POST programs in glufosinate-resistant soybean. Moreover, programs including three herbicide applications (PP fb PRE fb POST) did not result in fewer common ragweed or more biomass reduction, suggesting that PP fb POST effectively reduces common ragweed density. Aulakh and Jhala (2015) reported the greatest biomass reduction of broadleaf and grass weeds in glufosinate-resistant soybean with PRE fb POST compared to single or sequential POST programs. Similarly, Kaur et al. (2014) reported that herbicide programs with PP applications of 2,4-D, flumioxazin plus chlorimuron-ethyl, sulfentrazone plus cloransulam-methyl, or paraquat fb EPOST of glufosinate, alone or in tank-mixture, resulted in ≤14 plants m^−2^ and ≥88% biomass reduction of giant ragweed. The benefits of reducing GR common ragweed biomass and density extend into the following years as fewer GR common ragweed seeds can potentially enter the seed bank. The long survivability of common ragweed in the seed bank necessitates control measures that reduce the number of seeds returning to the seed bank each year.

Single application of PP herbicides in glufosinate-resistant soybean were unable to protect soybean yield potential, indicating that common ragweed can be extremely competitive in soybean fields if not controlled, or if controlled with only PP herbicide application without additional follow up treatments. Kaur et al. (2014) reported 100% soybean yield reduction when GR giant ragweed was not controlled. Similarly, Jhala et al. (2014) reported that a PP alone treatment resulted in 100% soybean yield reduction due to giant ragweed competition later in the season compared with PP fb POST programs. Furthermore, Aulakh and Jhala (2015) reported that a single POST herbicide application was ineffective in protecting soybean yield potential.

Gross profit margins were maximized with PP fb POST herbicide programs. Single applications resulted in low gross profit margin because of the inability of a single application to provide season-long control of common ragweed, thus allowing common ragweed to compete with soybean. Additionally, herbicide programs that included three applications protected soybean yield potential but the cost of herbicide and additional application cost reduced gross profit margins substantially. PP fb POST herbicide programs protected soybean yield potential and reduced the cost of application over other herbicide programs tested in this study.

## Conclusion

Results of this study conclude that PP herbicide options are available for early season control of GR common ragweed; however, a follow-up POST application of glufosinate alone or in tank-mixture is needed to achieve season-long control. Most of the PP herbicides tested in this study provided effective control (>95%) of common ragweed during soybean emergence and establishment. Furthermore, glufosinate can be used as an effective POST herbicide for control of GR common ragweed and can be tank-mixed with other herbicides such as *S*-metolachlor or acetochlor depending on the weed species present in the field. A recent update to the glufosinate (Liberty) label in the USA allows a maximum cumulative rate of 1,783 g ai ha^−1^ per growing season. Two applications, each of 656–881 g ai ha^−1^, could be made POST in glufosinate-resistant soybean before flowering (Anonymous, 2017). Soybean yields were reduced when a PP application was not made compared to PP fb POST programs, primarily due to early season common ragweed competition. PP fb PRE fb POST programs did not decrease density, improve biomass reduction, or increase soybean yield compared to PP fb POST programs, suggesting that three time herbicide application is not necessary for controlling GR common ragweed or protecting soybean yield.

The continued use of glufosinate can result in the evolution of glufosinate-resistant weeds through increased selection pressure; for example, Italian ryegrass (*Lolium perenne* L. ssp. *multiflorum* Lam. Husnot), perennial ryegrass (*Lomium perenne* L.), and goosegrass (*Galium indica* L. Gaertn.) have been documented resistant to glufosinate due to continuous use (Avila-Garcia and Mallory-Smith, 2011; Ghanizadeh et al., 2015; Jalaludin et al., 2015). Although, results of this study reported that herbicide options exist for control of common ragweed, an integrated weed management approach should be adopted for control of herbicide-resistant weeds that can include rotation of herbicides, the use of herbicides with multiple effective sites of action, the reintegration of tillage, and crop rotation.

## Author contributions

EB and AJ initiated research hypothesis and developed protocol; SK, PS, and JL Provided inout in the protocol; EB conducted research, analyzed data, and written manuscript. All coauthors provided input in the manuscript.

### Conflict of interest statement

The authors declare that the research was conducted in the absence of any commercial or financial relationships that could be construed as a potential conflict of interest.
